# Hypertrophic obstructive cardiomyopathy with recurrent ventricular tachycardias: from catheter ablation and stereotactic radiotherapy to heart transplant—a case report

**DOI:** 10.1093/ehjcr/ytae379

**Published:** 2024-07-30

**Authors:** Josef Kautzner, Jana Hašková, Jakub Cvek, Marek Adamíra, Petr Peichl

**Affiliations:** Department of Cardiology, Institute for Clinical and Experimental Medicine, Vídeňská 1958/9, 14300 Prague 4, Czech Republic; Department of Medicine 1, Palacky University Medical School, Palacky University Medical School Hospital, Zdravotníků 248/7, 77900 Olomouc, Czech Republic; Department of Cardiology, Institute for Clinical and Experimental Medicine, Vídeňská 1958/9, 14300 Prague 4, Czech Republic; Department of Medicine 1, Palacky University Medical School, Palacky University Medical School Hospital, Zdravotníků 248/7, 77900 Olomouc, Czech Republic; Department of Oncology, University Hospital Ostrava and Ostrava University Medical School, 17. listopadu 1790/5, 70800 Ostrava, Czech Republic; Department of Cardiothoracic Surgery, Institute for Clinical and Experimental Medicine, Prague, Czech Republic; Department of Cardiology, Institute for Clinical and Experimental Medicine, Vídeňská 1958/9, 14300 Prague 4, Czech Republic

**Keywords:** Catheter ablation, Hypertrophic cardiomyopathy, Stereotactic arrhythmia radioablation, Ventricular tachycardia, Electroanatomical mapping, Case report

## Abstract

**Background:**

Management of hypertrophic obstructive cardiomyopathy (HOCM) is often challenging, depending on clinical manifestation. This case report illustrates the complex treatment of HOCM with associated recurrent ventricular arrhythmias.

**Case summary:**

A 54-year-old female with HOCM diagnosed in 2012 underwent a failed attempt for alcohol septal ablation, implantation of an implantable cardioverter-defibrillator, and repeated radiofrequency ablations (including ablation of the septal bulge to reduce LV obstruction). For ventricular tachycardia (VT) recurrences, she had stereotactic arrhythmia radioablation with subsequent epicardial cryoablation from mini-thoracotomy, and endocardial ablation with pulsed field energy. The situation was finally solved by mechanical support and heart transplantation.

**Discussion:**

A few important lessons can be learned from the case. First, radiofrequency ablation was used successfully to decrease left outflow tract obstruction. Second, stereotactic radiotherapy has been used after four previous endo/epicardial catheter ablations to decrease the recurrences of VT. Third, mini-thoracotomy was used after previous epicardial ablation with subsequent adhesions to modify the epicardial substrate with cryoenergy. Fourth, pulsed field ablation of atrial fibrillation resulted in an excellent therapeutic effect. Fifth, pulsed field ablation was also used to modify the substrate for VT, and was complicated by transient AV block with haemodynamic deterioration requiring mechanical support. Finally, a heart transplant was the ultimate solution in the management of recurrent VT.

**Learning points**

- In hypertrophic obstructive cardiomyopathy, the leading presentation may be recurrent ventricular arrhythmias.
- Catheter ablation was used to moderate LV outflow tract obstruction and modify the substrate for arrhythmias.
- Despite repeated endo and epicardial ablations and stereotactic arrhythmia radioablation, heart transplant was indicated for ventricular tachycardia recurrences.

## Introduction

Hypertrophic cardiomyopathy (HCM) is an inherited cardiac condition (prevalence ≈ 1 in 500) associated with genetic and phenotypic heterogeneity.^1,2^ However, the disease-causing genes may remain unknown in nearly 25–40% of cases.^3,4^ It is characterized morphologically by an increased left ventricular wall thickness and mass and functionally by enhanced global chamber function and myocellular contractility, diastolic dysfunction, and myocardial fibrosis development. Significant pressure gradient in the left ventricular (LV) outflow tract can be measured in a variant with obstruction [hypertrophic obstructive cardiomyopathy (HOCM)]. Clinical manifestation varies from asymptomatic to sudden cardiac death. The most frequent symptoms include chest pain, exercise intolerance, and exertional dyspnoea. Management of HCM is often challenging, depending on clinical manifestation. This report illustrates the complex treatment of HOCM with recurrent ventricular arrhythmias.

## Summary figure

**Figure:**
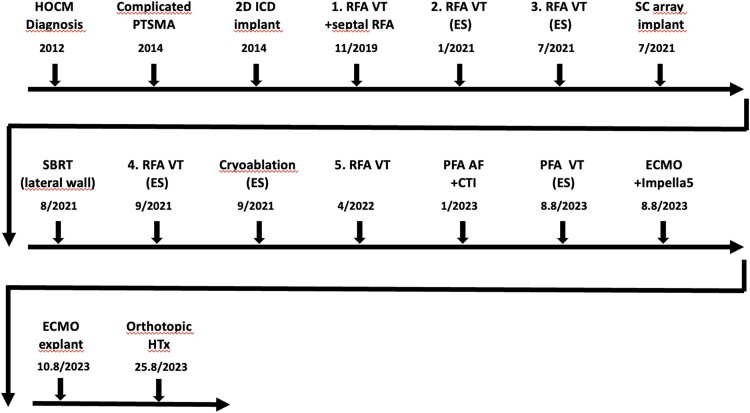


## Case presentation

**Figure 1:**
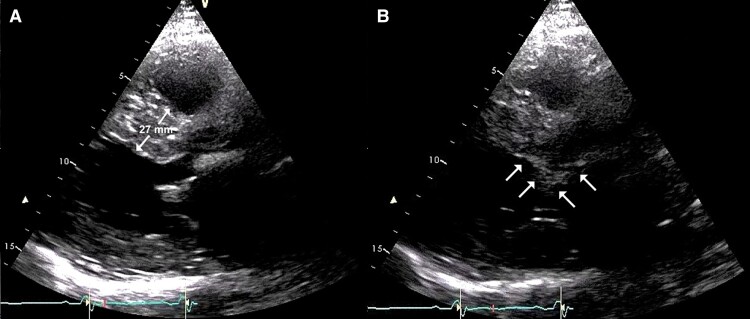
Echocardiogram shows (*A*) significant thickness of the interventicular septum and (*B*) systolic anterior motion of the anterior mitral leaflet (arrows).

**Figure 2:**
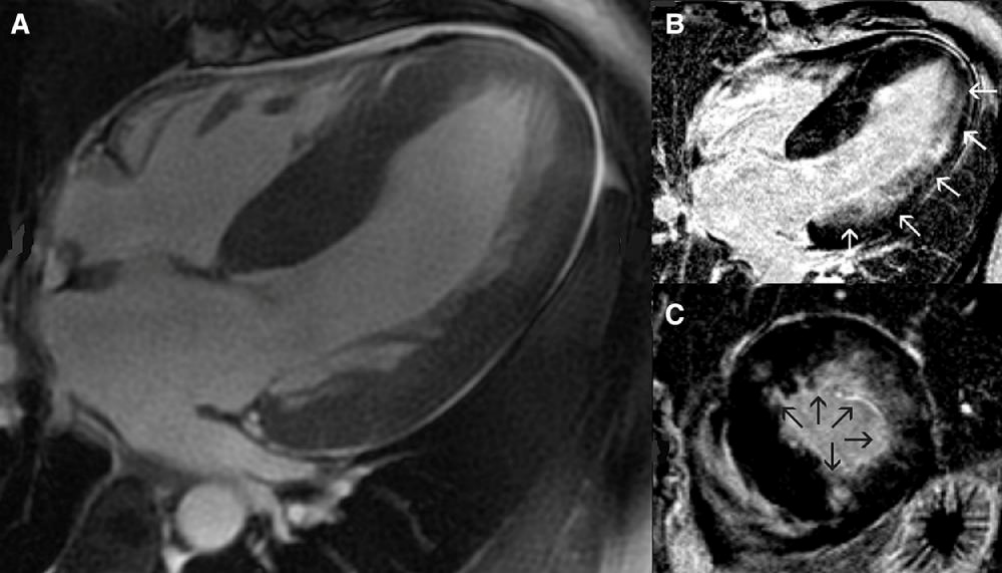
Magnetic resonance imaging depicting: (*A*) significant hypertrophy of the left ventricular walls. (*B*, *C*) Late gadolinium enhancement within the left ventricular wall (arrows).

The subject of this case report is a female patient born in 1970 without a family history of sudden cardiac death. In 2012, she was admitted for exertional dyspnoea (NYHA II–III) and suspected HOCM. Her body weight was 64 kg, her height 164 cm, and her BMI 23.2. Echocardiography showed significant hypertrophy of the interventricular septum (25–27 mm) and posterior wall (18 mm) with a resting pressure gradient in the LV outflow tract of 116 mmHg (*Figure 1*). The left atrium was dilated (left atrial volume index—LAVi 55 cm^3^/m^2^) with moderate mitral regurgitation. MRI confirmed HOCM with diffuse late enhancement, especially along the lateral wall, apicoseptally, and anterior wall (*Figure 2*). Whole exome sequencing did not reveal any gene associated with the disease. Biopsy and laboratory tests excluded common storage diseases.

The patient remained symptomatic on beta-blockers and underwent percutaneous transcoronary septal alcohol ablation in 2014. The procedure was complicated by acute dissection of the left anterior descending artery and its diagonal branch. Percutaneous coronary intervention with two drug-eluting stents solved the problem with minimal myocardial lesion (hs troponin up to 55 ng/L). However, alcohol ablation was abandoned. The proposed surgical myectomy was refused by the patient. In the next two years, a gradual drop in LV ejection fraction was observed to 40%. The calculated HCM risk score reached 5.26%, and the patient was implanted prophylactically with a dual chamber implantable cardioverter-defibrillator (ICD) in 2016. Pacing was optimized to maximize ventricular capture. Despite the improved quality of life (VO2 max 16.16 mL/min/kg, i.e. 75% of predicted value), the LV outflow gradient remained around 100 mmHg. LV ejection fraction further dropped to 30–35%. In 2019, she presented for the first time with an episode of atrial fibrillation and subsequently with an electrical storm with seven episodes of monomorphic ventricular tachycardia (VT).

**Figure 3:**
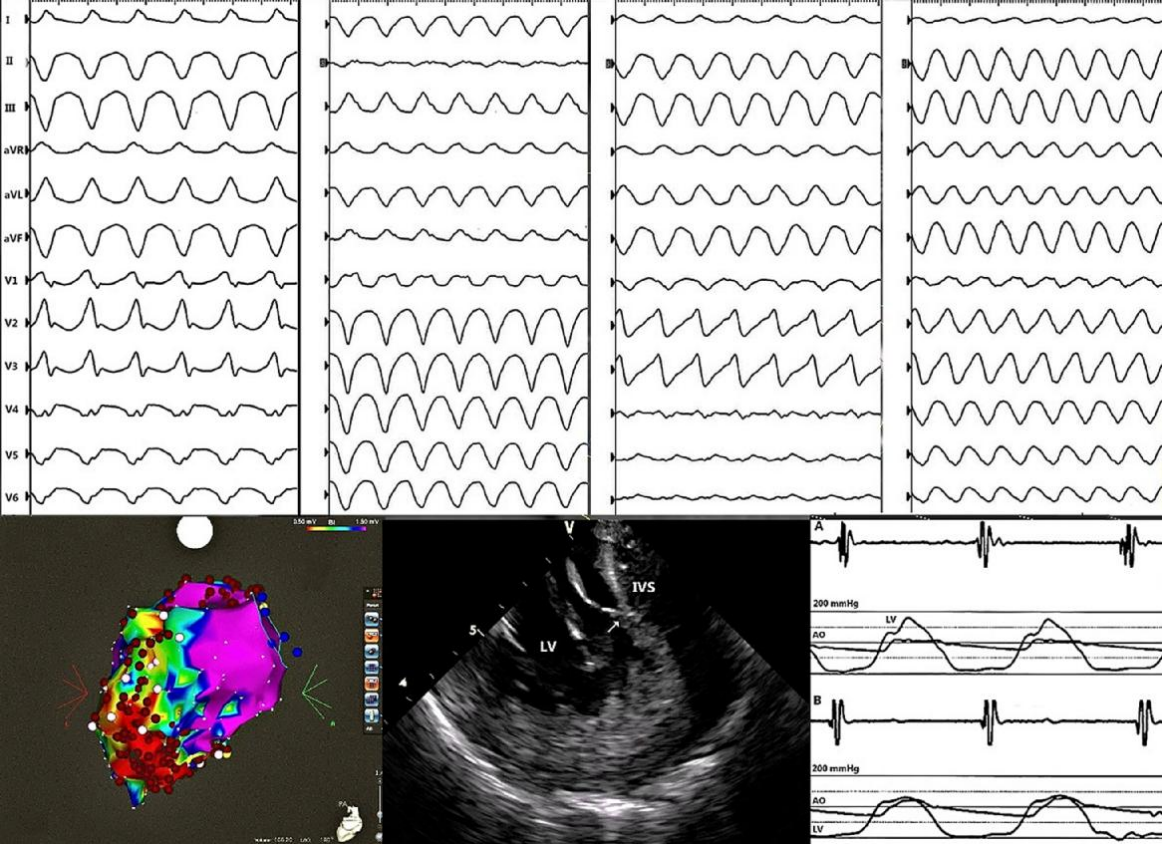
The upper panel shows ECG recordings of induced VTs during the first ablation. Lowe panel depicts (from left) the first electroanatomic map of the substrate, radiofrequency ablation on the septal bulge, and significant drop of the pressure gradient in the LV outflow tract.

**Figure 4:**
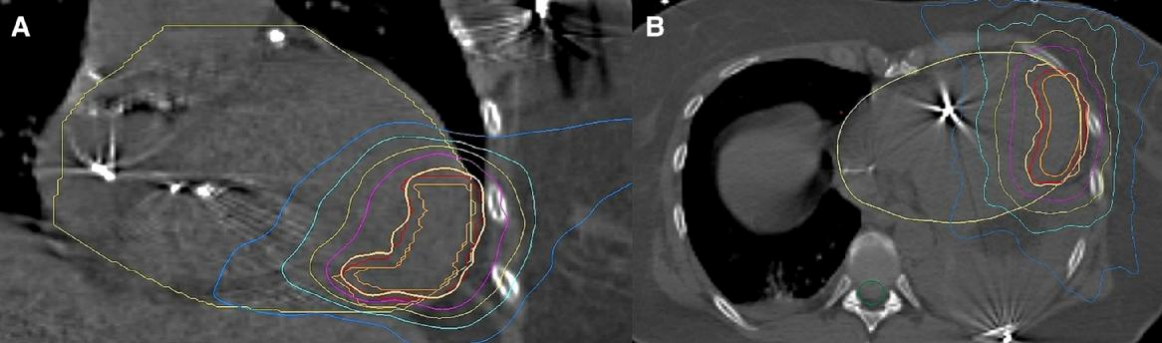
Radiotherapy treatment plan. Dose distribution in frontal (*A*) and axial (*B*) view. Clinical (orange) and planning (red) target volumes covered with prescribed isodose line 25 Gy. Purple, yellow, cyan, and blue lines represent 20, 15, 10, and 5 Gy, respectively.

Endocardial ventricular voltage mapping revealed a lateral wall scar extending to the inferior wall apically. Three monomorphic VTs were induced with inferoseptal (CL 314 and 290 ms) and apicolateral (CL 234 ms) exits. Only fast VT (CL 220 ms) remained inducible after extensive ablation of the substrate (*Figure 3*). Radiofrequency ablation on the septal bulging in the LV outflow tract was also performed. The resulting pressure gradient dropped significantly (*Figure 3*). Over a year of follow-up, the thickness of the interventricular septum decreased to 20 mm, and the pressure gradient further decreased (36 mmHg during the Valsalva manoeuvre). Despite symptomatic improvement, she was referred in January 2021 for another VT ablation due to an electrical storm (eight VT morphologies). In July 2021, epicardial mapping and ablation was performed for VT recurrences. Two VT morphologies were successfully ablated epicardially and five more endocardially, reaching a non-inducibility. In August 2021, she underwent a subcutaneous array implant due to ineffective ICD shocks, which resulted in an improvement of the defibrillation threshold. Subsequently, re-do ablation for an electrical storm was performed, targeting six VT morphologies from the apicoseptal to the anterolateral region. Due to recurrences of VT, she was indicated to stereotactic arrhythmia radioablation of the apical myocardial substrate (*Figure 4*). A single dose of 25 Gy was delivered using a robotic system (Cyberknife). Two weeks after radiotherapy, another re-ablation was required for incessant slow VT with an exit apicoseptally. The same month, a mini-thoracotomy was performed for recurrences of slow VT, and successful epicardial ablation was performed using cryoenergy. In April 2022, another endocardial ablation around the dense lateral scar was performed for recurrences of VT, again reaching non-inducibility.

**Figure 5:**
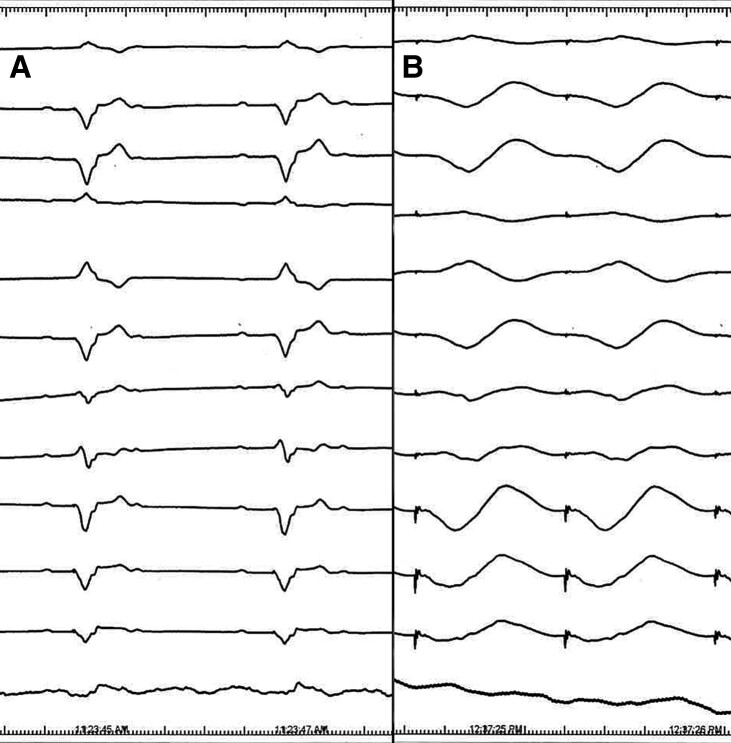
(*A*) Complete AV block as a transient side effect of pulse field therapy delivery (Centauri generator, Galvanize) through Smart Touch catheter (Biosense Webster). Further application resulted in very broad QRS complexes (QRS 410 ms) and haemodynamic deterioration. Leakage of the current through the proximal electrodes on the catheter shaft placed retrogradely in the left ventricle is a probable explanation for this complication.

In January 2023, she underwent complex ablation for recurrent atrial fibrillation (isolation of pulmonary veins, posterior wall, mitral, and cavotricuspid isthmuses) using a pentaspline catheter (Farawave, Boston Scientific) and pulsed field energy (PFE). No recurrences of atrial fibrillation were documented for 8 months. However, in August 2023, the patient was admitted for another electrical storm with four different VT morphologies. We decided to use PFE (Centauri generator, Galvanize) from an approved conventional irrigated tip catheter (SmartTouch, Biosense Webster). Lesions were delivered in four regions of the substrate, corresponding with exits of documented VTs. However, using a retrograde approach, the patient developed a transient AV block during PFE ablation on the lateral wall. After a series of PFE applications, bizarre broad QRS complexes appeared, and the patient became hypotensive with severe left ventricular dysfunction (*Figure 5*). She was intubated, and veno-arterial extracorporeal membrane oxygenation, and subsequently, Impella 5 (Abiomed, Inc.) were implanted, leading to haemodynamic stabilization. ECMO was explanted, and the patient underwent an orthotopic heart transplant without any complications. PFA lesions were clearly visible as rounded haemorrhagic necroses of 7–10 m diameter. Histology of the myocardium outside of ablated regions showed hypertrophic myocardial cells with nuclear enlargement, bizarre nuclei, fibre disarray, and interstitial fibrosis. Detailed description of the heart specimens and histology is beyond the scope of this report.

The patient had rapid postoperative recovery without complications. She overcame one episode of cellular rejection 1R/2 in November 2023 that was treated by Solumedrol. In March 2024, the patient was in a stable condition on immunosuppressive regimen of tacrolimus, mycophenolate mofetil, and prednisone. Echocardiography showed normal function of the graft.

## Discussion

This case documents all available treatment modalities in patients with the phenotype of HOCM and diffuse LV myocardial substrate for multiple VTs. A few important lessons can be learned from the case.

First, although transcoronary alcohol septal ablation is an established strategy for the management of LV outflow tract obstruction,^5^ radiofrequency septal ablation may help to decrease LV pressure gradient if other treatment modalities fail.^6,7^ In this case, radiofrequency ablation significantly reduced the pressure gradient and thickness of the septum. We believe that it might help to alleviate symptoms. Second, stereotactic arrhythmia radioablation has been used after four previous endo/epicardial catheter ablations to decrease the recurrences of VT. This strategy has the potential to decrease significantly the VT burden.^8,9^ Anecdotally, it was also used in HCM.^10^ In this patient, it was only partially successful. Third, mini-thoracotomy was used after previous epicardial ablation with subsequent adhesions to approach the substrate from epicardium with a cryoenergy. This is a well described alternative for such desperate cases.^11,12^ Despite acute success, even this strategy did not prevent recurrences of VT. Fourth, the case demonstrated potential of PFE through a pentaspline catheter to treat effectively atrial fibrillation in HCM. Ablation with radiofrequency current or cryoablation proved difficult in such patients.^13^ Fifth, we used PFE from conventional irrigated tip catheter in order to improve lesion penetration in scar regions.^14^ PFE has been reported anecdotally for VT ablation using different platforms. Having previous experience with Centauri generator (Galvanize) together with Smart Touch catheter (Biosense Webster) in VT ablation, we employed this technology. Interestingly, we observed an unexpected complication of PFE delivery. Our later observations suggest that this was due to a leakage of the current from the shaft electrodes of the ablation catheter that are used to display the shaft shape. This led to temporary AV block and haemodynamic deterioration, necessitating the use of mechanical heart support. Finally, a heart transplant was the ultimate solution in the management of recurrent VT in this patient with non-isomeric HCM. Such an option has been described previously.^15^

## Conclusions

This case of HOCM with a diffuse LV myocardial substrate for multiple VTs and recurrent electrical storms required multiple treatment strategies: specifically, repeated radiofrequency ablation (including ablation of the septal bulge to reduce LV obstruction), stereotactic radiotherapy, cryoablation, ablation with PFE, and ultimately, heart transplantation. We believe that repeated ablation procedures, including ablation of the septal bulge and ablation of atrial fibrillation, helped to slow down the progression of the disease to the terminal stage.

## Data Availability

We describe clinical case where most of all available data are presented within the manuscript. All ablations reports, electrograms, electroanatomical maps, and images are available in digital format in our hospital information system.

## Lead author biography

Prof. Josef Kautzner, MD, PhD, graduated at Charles University in Prague and obtained a substantial part of his training at the Department of Medicine II, Charles University General Hospital and during stays in London or Houston. He has been working at the Institute for Clinical and Experimental Medicine in Prague since 1996, currently as a Director of the Heart Centre. His clinical interest is focused mainly on cardiac arrhythmias and imaging. He is an author of four books, many book chapters, and authored or co-authored more than 400 scientific papers. H index in January 2024 reached 70 with 30 414 quotations.

## Notes

**Consent:** We hereby confirm compliance with the COPE guidelines and received written consent from the patient about publication of the case report.

**Funding:** This study was supported by the project National Institute for Research of Metabolic and Cardiovascular Diseases (Programme EXCELES, project no. LX22NPO5104)—funded by the European Union—Next Generation EU and by funding from the European Union’s Horizon 2020 research and innovation programme under grant agreement no. 945119 (STOPSTORM).
